# Kill two birds with one stone: Flexible cholangioscopy for treatment of common bile duct stone and identification of suspicious gallbladder wall thickening

**DOI:** 10.1055/a-2299-2307

**Published:** 2024-04-24

**Authors:** Ruixin Zhang, Tao Yu, Rui Ji

**Affiliations:** 1 91623 Department of Gastroenterology, Qilu Hospital of Shandong University, Jinan, China

A 76-year-old man suffered from recurrent abdominal pain and distention. The contract-enhanced computed tomography (CT) and endoscopic ultrasound (EUS) exams revealed the presence of a stone in common bile duct (CBD) ( Fig. 1 **a** ) and concerning evidence of gallbladder wall thickening, suggestive of either gallbladder adenomyosis or gallbladder carcinoma ( Fig. 1 **b** , **c** ). In order to distinguish benign adenomyosis from malignant carcinoma, together with treatment of the CBD stone, we sequentially performed endoscopic basket retrieval and target biopsies of the gallbladder.

**Fig. 1:**
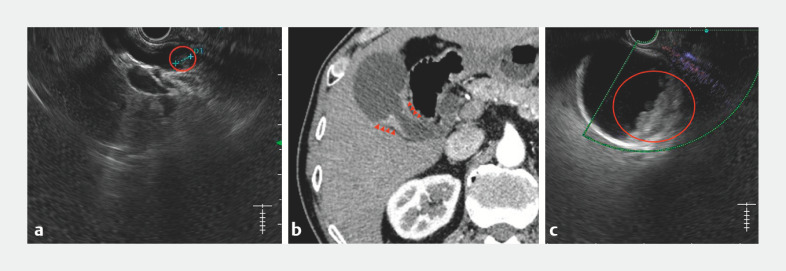
**a** Endoscopic ultrasound showed the stone in the common bile duct (CBD) (red circle). **b** Contrast-enhanced abdominal computed tomography revealed the contrast-enhanced localized thickening of the gallbladder wall (red arrows). **c** The localized contrast-enhanced gallbladder wall thickness as shown in the endoscopic ultrasound image (red circle).

In the first procedure, contrast imaging revealed CBD dilation ( Fig. 2 **a** ). Under the navigation of the guidewire, the cholangioscope was introduced into the CBD and the stone was then successfully extracted ( Fig. 2 **b** ). Subsequently, another guidewire was advanced into the gallbladder lumen under the guidance of the cholangioscope ( Fig. 2 **c** ), facilitating the subsequent placement of the stent along the segment extending from the gallbladder to Vater’s ampulla, creating an approach for subsequent cholangioscopy during the second procedure ( Fig. 2 **d** ). Meanwhile, a double-pigtail stent was deployed within the CBD to ensure fluent bile drainage.

**Fig. 2:**
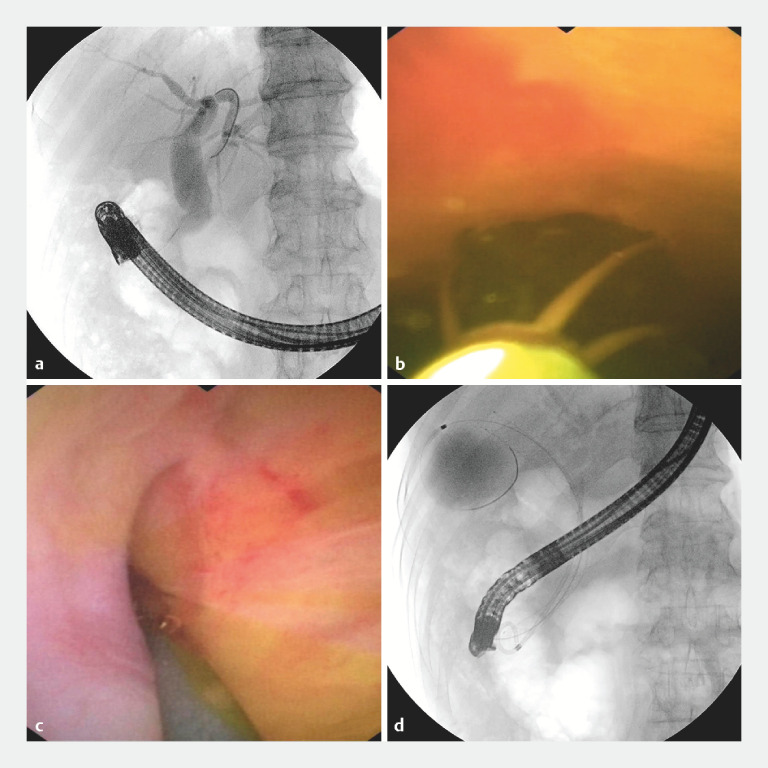
**a** Cholangiography showed the dilation of CBD. **b** The stone was extracted by the retrieval basket. **c** The guidewire was advanced into the gallbladder lumen under direct vision of the cholangioscope. **d** Cholangiography showed the placement of the metal stent and double-pigtail stent.

Three days later, we performed the second endoscopic retrograde cholangiopancreatography (ERCP). The cholangioscope entered the gallbladder along the formally established pathway under the guidance of the guidewire. Owing to the excellent dexterity of the cholangioscope, retroflexion can be achieved in the constricted gallbladder lumen, so that we could directly observe the multifocal papillary neoplasm at the gallbladder neck ( Fig. 3 **a** , **b** ). Subsequently, target biopsies were taken and then pathology confirmed high-grade intraepithelial neoplasia. Stents were removed during duodenoscope withdrawal ( Fig. 3 **c** , **d** ; Video 1 ).

**Fig. 3:**
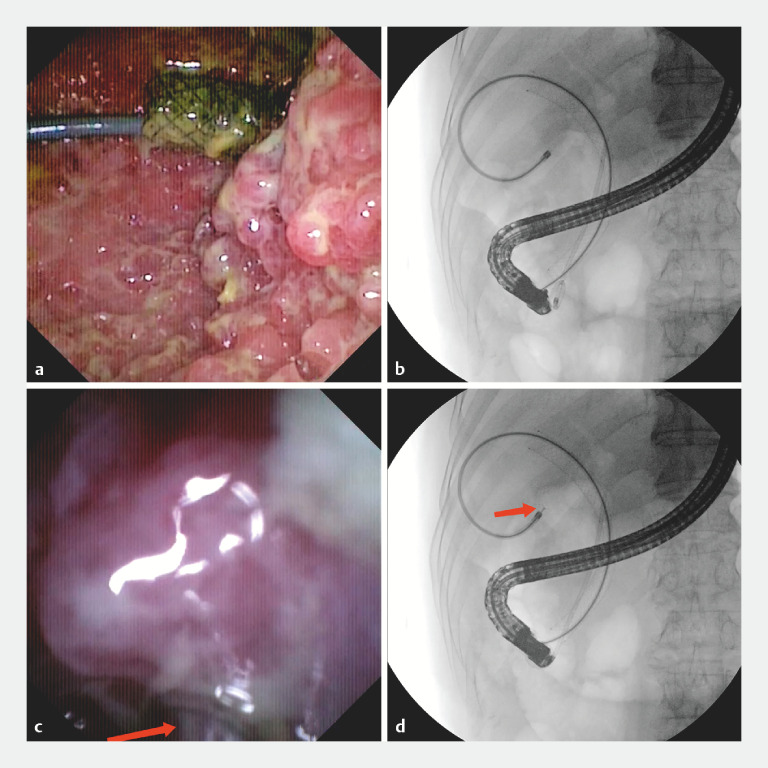
**a** Multifocal irregular papillary proliferations were observed at the gallbladder neck in retroflexion. **b** Contrast imaging presented the cholangioscope in the retroflex position. **c** Target biopsies were taken by forceps. **d** Contrast imaging presented the process of the biopsy.

Cholangioscopy-guided stone extraction and biopsy.Video 1

The patient then underwent laparoscopic cholecystectomy. Postoperative histopathology confirmed the presence of highly differentiated gallbladder adenocarcinoma.

As this case shows, while gallbladder neoplasms can be easily detected by US or CT, making a pathological diagnosis in the early stage remains challenging, often necessitating endoscopic ultrasound-guided fine needle aspiration (EUS-FNA) 1 . This case suggests a potential option for early precise diagnosis of a gallbladder neoplasm.

Endoscopy_UCTN_Code_TTT_1AR_2AD

## Notes

Endoscopy E-Videos https://eref.thieme.de/e-videos
*E-Videos* is an open access online section of the journal *Endoscopy* , reporting on interesting cases and new techniques in gastroenterological endoscopy. All papers include a high-quality video and are published with a Creative Commons CC-BY license. Endoscopy E-Videos qualify for HINARI discounts and waivers and eligibility is automatically checked during the submission process. We grant 100% waivers to articles whose corresponding authors are based in Group A countries and 50% waivers to those who are based in Group B countries as classified by Research4Life (see: https://www.research4life.org/access/eligibility/ ). This section has its own submission website at https://mc.manuscriptcentral.com/e-videos .
